# Cytotoxic Granule Trafficking and Fusion in Synaptotagmin7-Deficient Cytotoxic T Lymphocytes

**DOI:** 10.3389/fimmu.2020.01080

**Published:** 2020-05-29

**Authors:** Marwa Sleiman, David R. Stevens, Praneeth Chitirala, Jens Rettig

**Affiliations:** Cellular Neurophysiology, Center for Integrative Physiology and Molecular Medicine (CIPMM), Saarland University, Homburg, Germany

**Keywords:** cytotoxic T lymphocytes, cytotoxic granules, granule fusion, granule trafficking, immune synapse, calcium dependence, synaptotagmin

## Abstract

Granules of cytotoxic T lymphocytes (CTL) are derived from the lysosomal compartment. Synaptotagmin7 (Syt7) appears to be the calcium sensor triggering fusion of lysosomes in fibroblasts. Syt7 has been proposed to control cytotoxic granule (CG) fusion in lymphocytes and mice lacking Syt7 have reduced ability to clear infections. However, fusion of CG persists in the absence of Syt7. To clarify the role of Syt7 in CTL function, we have examined the fusion of cytotoxic granules of CD8^+^ T-lymphocytes from Syt7 knock-out mice. We have recorded granule fusion in living CTL, using total internal reflection microscopy. Since Syt7 is considered a high affinity calcium-sensor specialized for fusion under low calcium conditions, we have compared cytotoxic granule fusion under low and high calcium conditions in the same CTL. There was no difference in latencies or numbers of fusion events per CTL under low-calcium conditions, indicating that Syt7 is not required for cytotoxic granule fusion. A deficit of fusion in Syt7 KO CTL was seen when a high-calcium solution was introduced. Expressing wild type Syt7 in Syt7 KO lymphocytes reversed this deficit, confirming its Syt7-dependence. Mutations of Syt7 which disrupt calcium binding to its C2A domain reduced the efficacy of this rescue. We counted the cytotoxic granules present at the plasma membrane to determine if the lack of fusion events in the Syt7 KO CTL was due to a lack of granules. In low calcium there were no differences in fusion events per CTL, and granule numbers were similar. In high calcium, granule number was similar though wild type CTL exhibited significantly more fusion than Syt7 KO CTL. The modest differences in granule counts do not account for the lack of fusion in high calcium in Syt7 KO CTL. In Syt7 KO CTL expressing wild type Syt7, delivery of cytotoxic granules to the plasma membrane was comparable to that of wild type CTL. Syt7 KO CTL expressing Syt7 with deficient calcium binding in the C2A domain had significantly less fusion and fewer CG at the plasma membrane. These results indicate that Syt7 is involved in trafficking of CG to the plasma membrane.

## Introduction

Cytotoxic T lymphocytes (CTL) play an important role in the adaptive cellular immune response by killing virally infected cells and tumor cells (1). This involves the release of cytotoxic substances from cytotoxic granules (CG) via exocytosis at a CTL-target cell contact area, the immune synapse (IS) (2). Exocytosis requires fusion of the CG membrane with the plasma membrane, an event driven by a protein complex (soluble NSF attachment receptor (SNARE) complex) consisting of four coiled-coil protein domains contributed by three proteins referred to as SNARE proteins (3).

SNARE complexes utilize a highly conserved set of homologous proteins required for virtually all fusion reactions involving two lipid membranes (4). A highly regulated form of exocytosis triggered by increases in intracellular [Ca^2+^] requires, in addition to the SNARE proteins, Ca^2+^-sensing synaptotagmins (Syt) (5). Synaptotagmins are predominantly found in neuronal cells, but are present in many other cell types where they also function as Ca^2+^ sensors and may prevent fusion of assembled SNARE complexes in the absence of a Ca^2+^ stimulus (6).

CG are considered to be hybrid granules which exhibit the properties of both lysosomes and secretory granules (7–10). CG express the lysosomal membrane markers LAMP1 and LAMP2 and contain lysosomal proteolytic enzymes which are maintained in an acidic lumenal environment. CG are generated via the endosomal/lysosomal compartment (11), and appear to be the result of the fusion of lysosomes with vesicles in which the cytotoxic proteins granzyme A, granzyme B and perforin are stored (12).

Fusion of lysosomes with the plasma membrane is Ca^2+^-dependent and utilizes synaptotagmin7 (Syt7) as its Ca^2+^ sensor (13). Syt7 has also been reported to be expressed and to function in CG fusion in CTL (14). Syt7-deficient CTL are less effective at pathogen clearance and are defective in killing target cells in a peptide-specific killing assay, in spite of the fact that immune synapse formation and granule polarization appear normal. Since there are alternative pathways to target cell elimination, immune function in the absence of Syt7 may occur in spite of a CG fusion deficit, but this seems unlikely since granzyme release following CD3^+^ crosslinking *in vitro* was not reduced in the Syt7 KO cells (14). Syt7 has a high Ca^2+^ affinity when compared to other synaptotagmins and thus may be particularly suited for fusion of CGs associated with relatively low intracellular Ca^2+^ levels (5). Syt7 also functions in cell migration (15, 16) and membrane repair (14, 15, 17), processes which also involve fusion of vesicles with the plasma membrane. Exocytosis of lysosomes as well as CG fusion occur at intracellular free [Ca^2+^] of 1–5 μM, though target cell killing has been observed at lower Ca^2+^ levels in some experiments (18). We have examined CG exocytosis in mouse CD8^+^ lymphocytes using live-cell imaging following anti-CD3 antibody stimulation in wild type and Syt7-deficient CTL in order to determine whether CG fusion occurs in the absence of Syt7 and to better understand the role of Syt7 in CTL function. Our results indicate that Syt7 is not required for CG fusion, but plays an important role in trafficking of CGs to the immune synapse.

## Materials and Methods

### Mice

C57BL6/N and Syt7 KO mice from Jackson Laboratory were used in all experiments. All experimental procedures were approved and performed according to German federal regulations and to regulations of Saarland.

### Cell Culture

Splenocytes were isolated from 8 to 12 week-old synaptotagmin7 knock-out (Syt7 KO) or C57BL6/N mice, as described before (19). Briefly, CD8^+^ T cells were positively isolated from splenocytes using the Dynabeads FlowComp Mouse CD8^+^ kit (Fisher Scientific) according to the manufacturer's instructions. The isolated CD8^+^ T cells were activated with mouse anti-CD3/anti-CD28 (1:0.8 ratio) and cultured in IMDM medium (Iscove Modified Dulbecco Medium, Invitrogen) containing 10% FCS, 0.5% pen/strep and 50 μM 2-mercaptoethanol at a density 1 × 10^6^/mL in a 24-well plate for 2 days at 37°C with 5% CO_2_.

### Nucleofection of Expression Constructs and Silencing of Gene Expression by siRNA

After 2 days of activation CTL were transferred to a 12-well plate and supplemented with fresh IMDM medium and mouse IL-2 (50 U/mL). 5 × 10^6^ cells were transfected with 1 μg of plasmid DNA (Amaxa^TM^ Mouse T cell Nucleofector Kit, Lonza). Seventy to eighty per cent of cells were viable after transfection, the transfection efficiency was 32.4 ± 12.2% (mean ± SD). Cells were seeded in a 24-well plate under normal culture conditions as described before and measured on day 3, 12–16 h after transfection. For silencing of synaptotagmin2 expression cells were transfected with siRNA1 5′-ATG GAT GGT GTT GTA GAG TTT-3′, siRNA2 5′-ACC GTG CTA GAC TAC GAC AAA-3′ and negative control siRNA (Qiagen) at a final concentration of 20 μM for each siRNA. Cells were harvested for RNA isolation 24–30 h after transfection.

### RNA Isolation, Reverse Transcription and PCR

Total RNA was extracted from 5 × 10^6^ CTL and total mouse brain with TRIzol (Thermo Fisher Scientific) and reverse transcribed with SuperScript II (Thermo Fisher Scientific) using random hexamer primers (Invitrogen). Semi-quantitative PCR was performed with 100 ng of CTL and 25 ng brain cDNA using specific intron-spanning primers as described in Table S1.

### DNA Constructs

Syt7α was amplified from total brain cDNA (forward primer: 5′-TAT AGG ATC CGC CAC CAT GTA CCC ATA CGA TGT TCC AGA TTA CGC TTA CCG GGA CCC GGA CGC G-3′; reverse primer: 5′-TAT AGC GCG CTC AGG CTT TCA GCT GGT GCC-3′) and cloned into a pMAX-IRES-GFP vector. The single (D227, forward primer: 5′-CTG GAT TAT AAC CGT TTC AGC-3′; reverse primer: 5′-GCT GAA ACG GTT ATA ATC CAG-3′) and triple aspartate (D225N, forward primer: 5′-CAG GTC CTG AAT TAT GAC CG-3′, reverse primer: 5′-CGG TCA TAA TTC AGG ACC TG-3′; D233N, forward primer: 5′-CAG CCG CAA TAA CCC CAT TGG-3′, reverse primer: 5′-CCA ATG GGG TTA TTG CGG CTG-3′) to asparagine mutants were generated by PCR using the Syt7α-pMAX-IRES-GFP vector as template. The pMAX-granzyme B-mCherry construct was generated as described before (20).

### Immunofluorescence and Structured Illumination Microscopy (SIM)

Three days after activation CTL were transfected with Syt7-IRES-eGFP and granzyme B-mCherry. Eight hours after transfection, cells were seeded onto anti-CD3e (Cell sciences) coated coverslips and fixed with freshly prepared, ice-cold PFA (4%). Cells were washed with PBS and permeabilized by 0.1% Triton X-100 (Roth) in D-PBS (Invitrogen) for 20 min at RT. After blocking in D-PBS with 0.1% Triton X-100 and 2% BSA for 30 min cells were stained with rabbit anti-Syt7 primary antibody (Synaptic Systems, 1:200) and goat anti-rabbit antibody conjugated with Alexa405 (Life Technologies, 1:2000) and mounted for imaging. To compare the localization of Syt7 and CD3, a rat anti-CD3-Alexa647 primary antibody (BD Biosciences, 1:200) was used under the same conditions. Cells were imaged using high-resolution structured illumination microscopy (SIM) from Zeiss (ELYRA PS.1; Carl Zeiss Microscopy GmbH). Images were acquired by using Zen2012 software (Carl Zeiss Microscopy GmbH) and a 63x Plan-Apochromat (NA 1.4) objective with excitation light of 405, 488, 561, and 647 nm wavelengths and processed for higher resolution. Cells were imaged using ZEN software after acquiring multiple stacks with a step size of 200 nm.

### Western Blotting

To detect Syt2 expression levels, un-transfected CTL and siRNA-treated cells were lysed by sonication in lysis buffer (50 mM Tris (pH 7.4), 150 mM NaCl, 1 mM EDTA, 1 mM deoxycholate, 1 mM DTT, 1% Triton X-100, 200 μM PMSF and protease inhibitors) on ice. Lysates were rotated for 30 min at 4°C and the supernatant was collected after 10 min centrifugation at 15.000 x *g*. Protein concentrations were measured with Bradford Assay (Bio-Rad). Proteins were separated by SDS-PAGE on 4–12% gradient Bis-Tris gels (Thermo Fisher Scientific), transferred to nitrocellulose membrane (0.45 μm, Thermo Fisher Scientific) and blocked with 5% non-fat dry milk in TBS buffer containing 20 mM Tris, 0.15 M NaCl and 0.1% Tween 20, pH 7.4 for 1.5 h at RT. The membrane was blotted with rabbit anti-Syt2 antibody (Abcam, 1:1000) and rabbit anti-GAPDH antibody (Cell Signaling, 1:5000) overnight at 4°C. After incubation with HRP-conjugated goat anti-rabbit secondary antibody (Millipore Sigma, 1:5000) the blot was developed by using enhanced chemiluminescence reagents (SuperSignal West Dura Chemiluminescent Substrate, Thermo Fisher Scientific) and imaged by gel documentation (FluorChem E system, BioLabTec).

### Total Internal Reflection Fluorescence (TIRF) Microscopy

Measurement of CG fusion was carried out on a commercial TIRF setup from Visitron Systems (Puchheim, Germany) composed of an IX83 inverted microscope equipped with autofocus module, a UAPON100XOTIRF NA 1.49 objective (all from Olympus), the iLAS^2^ beam scanning system (Gataca Systems, Paris, France), an evolve-EM 515 EMCCD camera (Photometrics, Tucson, AZ, USA), a 488 nm 100 mW laser (Toptica) and a filter cube housing a ZT405/488/561/640rpc multi-band dichroic and ZET405/488/561/640rpc multi-band emission filter (Semrock). All setup components were controlled by VisiView (Visitron Systems GmbH). The final pixel size was 0.16 μm^2^. Illumination angle was 67.5° leading to a penetration depth [δ*(*θ*)*] of the evanescent wave of 134 nm (21). Because δ*(*θ*)* is the distance over which the light intensity drops to 1/e (37%) of its value at the interface CGs are visible within ~300 nm of the plasma membrane attached to the glass coverslip. The frame acquisition rate was 10 Hz and the exposure time was 100 ms.

Granule fusion analysis was begun immediately after plating of CTL. Fusion events were recorded in low calcium for 7 min (a nominally calcium free solution [NaCl 155 mM, KCl 4.5 mM, Hepes 5 mM, MgCl_2_ 3 mM, glucose 10 mM, 300–310 mOsm/l, pH = 7.4]). The recording chamber was then flooded with a high calcium solution (NaCl 140 mM; KCl 4.5 mM; Hepes 5 mM; MgCl_2_ 2 mM; CaCl_2_ 10 mM; glucose 10 mM, 300–310 mOsm/L, pH = 7.4) and fusion events were recorded for an additional 7 min. In a set of control recordings the low calcium solution was applied for the entire 14 min recording period.

### Measurement of Intracellular Free Ca^2+^ Using Fura-2 AM

The ratiometric Ca^2+^ indicator Fura-2 AM, illuminated at 350 nm and 380 nm excitation wavelengths for the Ca^2+^-bound and Ca^2+^-free forms of Fura-2, was used. The ratio of the emission fluorescence intensities after excitation at these wavelengths was calculated to determine the real [Ca^2+^]_i_ (22). Fura-2 calibration solutions at R_min_ (~0 nM), R_max_ (10 mM) and R (268 nM) were applied via patch pipette to Jurkat cells. To determine the concentration of free Ca^2+^ in WT and Syt7 KO cells in the TIRF experiments, CTL 3 days after activation were collected and the activating magnetic beads removed. CTL were washed in PBS and re-suspended in 500 μL of 2 μM Fura-2 AM medium (Fura-2 AM + serum-free IMDM medium). Cells were incubated with Fura-2 AM for 30 min at RT and washed afterwards twice with IMDM medium. After washing, the pellet was resuspended in 0 mM Ca^2+^ Ringer's solution and CTL were placed on anti-CD3 coated glass coverslips. CTL were observed under the same conditions as in the TIRF experiments.

### Measurement of Extracellular Free Ca^2+^ Using Fura-2ff

To determine the concentration of extracellular Ca^2+^ under low Ca^2+^ measurement conditions, CTL 3 days after activation were pelleted, the supernatants collected and Fura-2ff (100 μM) was added. 1.5 μL of supernatant was pipetted on to a coverslip and measured ratiometrically (at 350 and 380 nm). This procedure was repeated three times. Six different calibration solutions with free [Ca^2+^] of 10 nM, 1.33 μM, 23.4 μM, 108 μM, 518 μM, and 9.4 mM were prepared. The emitted fluorescence intensity of Fura-2ff when excited at 350 nm and 380 nm was measured and the 350/380 ratio as a function of the known concentrations plotted. The points were fit with a Hill curve and the free [Ca^2+^]_o_ was estimated from this curve.

### Statistical Analysis

Statistical analysis and data plotting was carried out using Igor Pro. When not otherwise stated, comparisons were made using the Wilcoxon Rank test carried out in Igor Pro. In some cases the Student's *t*-test was applied to compare samples whose distributions were normal. *P*-values are presented when significant. *P*-values lower than 0.001 are presented as *p* < 0.001.

## Results

### Synaptotagmin2 and Synaptotagmin7 Are the Only Ca^2+^-Sensitive Paralogs Expressed in CTL

**Figure 1:**
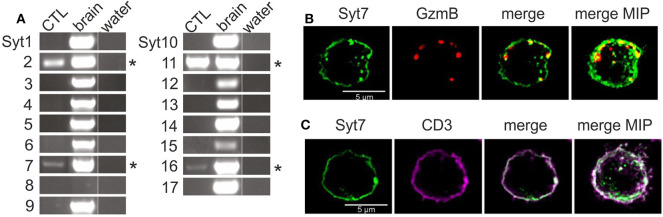
The Ca^2+^-sensitive synaptotagmins 2 and 7 are expressed in CD8^+^ mouse CTL. **(A)** Expression analysis of the 17 known synaptotagmins in activated mouse CTL and mouse brain by semi-quantitative RT-PCR (*n* = 3). Synaptotagmins 2, 7, 11, and 16 were expressed in activated CTL, with Syt7 and Syt16 exhibiting relatively weak expression. For details of PCR conditions see also Table S1. **(B)** Exogenously expressed synaptotagmin7 exhibits punctate and membrane-like staining. Representative SIM images of activated CTL expressing granzyme B mCherry and Syt7-IRES-GFP after fixation and staining with an antibody against Syt7. Some Syt7 (green, Alexa 405). puncta overlap the signal from the granzyme B-mCherry fluorescence (red) in the merged. **(C)** Peripheral Syt7 staining is indeed membrane staining. Representative SIM images show plasma membrane staining with an anti-CD3 antibody (violet) and staining with anti-Syt7 (green).

We tested for the presence of RNA of all 17 synaptotagmin paralogs by paralog-specific oligonucleotides (Table S1) using RT-PCR. Four paralogs, Syt 2, 7, 11, and 16 (Figure 1A, asterisks) were detected in lysate from mouse CTL (left lane) on day 3 of activation, while all paralogs except Syt8 could be detected in RNA extracted from mouse brain (center lane, Figure 1A). Of the four detected paralogs, Syt16 and Syt11 were excluded as candidate Ca^2+^ sensors due to their lack of consensus Ca^2+^ binding sites (5). The remaining synaptotagmins, Syt2 and Syt7, are established Ca^2+^ sensors in neuronal and (neuro)endocrine cells (23–25). Though Syt2 was more abundant at the RNA level, Syt7 has been implicated in fusion of lysosomes in fibroblasts and in fusion CGs in CTL (see above). A further argument in favor of Syt7 is its high Ca^2+^ affinity (discussed above), because target killing by CG exocytosis can occur at low intracellular Ca^2+^ levels (18).

We tested for expression of Syt7 and Syt2 protein by Western blot using commercially available antibodies in CTL 3 days after bead stimulation. Syt2 was detected in wild type CTL and in Syt7 KO CTL (Figure S1). Although Syt7 expression has been reported in activated mouse CTL (14) we did not detect it in Western blots. This discrepancy likely is due to low protein abundance but may be due to the antibody used.

Based on reports of a role for Syt7 in the fusion of lysosomes and CG of CTL (see above), we used immunostaining to determine whether Syt7 associated with CGs. Since endogenous Syt7 was not detected, CTL were electroporated with two cDNA constructs, coding for a granzyme B-mCherry fusion protein and Syt7-IRES-GFP, respectively. After 8 h the cells were fixed, permeabilized, treated with an antibody against Syt7 (see Materials and Methods) and observed using SIM (structured illumination microscopy) (Figure 1B). Syt7 staining was present as granular structures and as membrane-like staining in single 0.2 μm thick z-sections. Granzyme B-mCherry (GzmB) fluorescence was exclusively punctate consistent with its presence in CG. Both merged image and maximum intensity projection (far right) showed modest co-localization of GzmB with some Syt7 puncta (yellow, 0.198 ± 0.048, Manders' coefficient, overlap Syt7 to GzmB, 0.292 ± 0.058, Manders' coefficient, overlap of GzmB to Syt7, *n* = 13). Apparent Syt7 membrane staining was detected as expected, since after exocytosis Syt7 associated with CGs will remain at the plasma membrane until endocytosis returns it to the cytoplasm, and Syt7 has been reported to bind to the plasma membrane in a number of cell types [discussed in (26)].

To confirm that non-granular Syt7 staining is indeed at the plasma membrane, we compared immunostaining of Syt7 to that of CD3, a plasma membrane marker in CTL. Figure 1C shows a SIM image of a representative CTL immuno-stained for both Syt7 and CD3. Syt7 staining (green) was punctate with some membrane-like staining. CD3 (magenta) exhibited membrane staining with a few punctate structures which were also at the plasma membrane. In the merged image there is co-localization (white, Manders' coefficient of correlation: 0.61 ± 0.043, *n* = 13) at the plasma membrane. On the far right is a merged MIP showing a similar result.

### Initial CG Fusion in Synaptotagmin7-Deficient CTL Is Unaltered, but CG Replenishment Is Reduced

We then compared CG fusion in mouse CD8^+^ CTL from Syt7 KO mice with that of wild type CTL under conditions of low and high extracellular [Ca^2+^]. CTL derived from wild type or Syt7 KO mice with the same genetic background (C57Bl/6N) were plated on anti-CD3 treated coverslips in a nominally zero-calcium [low [Ca^2+^]] solution. The cells had been electroporated with the granzyme B-mCherry construct which exhibits red fluorescence when illuminated at 561 nm.

**Figure 2:**
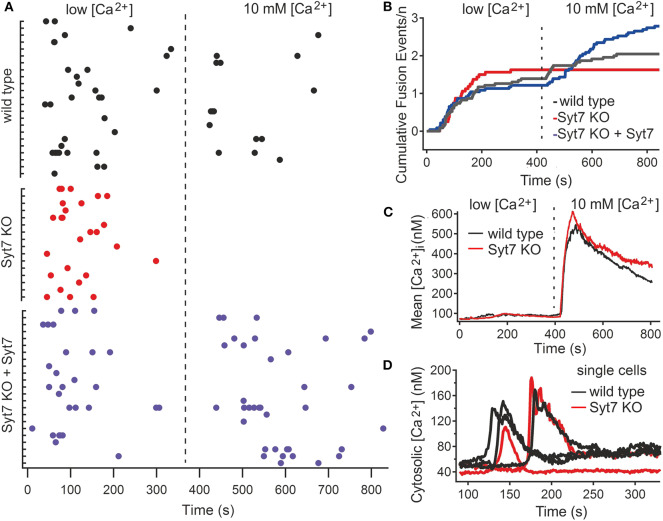
Syt7 is required for a late phase of CG fusion. **(A)** Scatter plots showing CG fusion events for all CTL (black, wild type (*n* = 23); red, Syt7 KO (*n* = 16) and blue, Syt7 KO expressing Syt7 (*n* = 23). The cells are grouped into wild type and Syt7 KO CTL, with each tic on the Ordinate representing a single CTL and the Abscissa representing time, with the dashed line indicating the change from low [Ca^2+^] to 10 mM [Ca^2+^]. Each dot represents a fusion event. Each group exhibited similar fusion in low [Ca^2+^] (blue). **(B)** Plots of cumulative fusion vs. time for the 14 min observation periods show that fusion was similar in wild type (black), Syt7 KO (red), and Syt7 KO expressing Syt7 cells in the first 7 min. **(C)** The mean cytosolic calcium concentration is similar in wild type (*n* = 34) and Syt7 KO (*n* = 31) under the conditions of our experiments. Upon the change to 10 mM [Ca^2+^] a similar increase in cytosolic calcium was observed wild type and Syt7 KO cells. **(D)** In wild type (*n* = 17) and Syt7 KO (*n* = 14) CTL, transient increases in cytosolic calcium associated with store release are of similar amplitude and duration.

CG fusion in total internal reflection fluorescence microscopy (TIRFM) was identified by a rapid decrease of mCherry fluorescence, associated with a rapidly dispersing cloud of fluorescence (see Figure S2). CG fusion occurred in both wild type (*N* = 3, *n* = 23) and Syt7 KO (*N* = 3, *n* = 16) CTL in the low [Ca^2+^] period with most events occurring in the first 3 min, demonstrating that Syt7 is not required *per se* for granule fusion (Figure 2A). In the low [Ca^2+^] solution the fraction of CTL exhibiting exocytosis was similar for control (18.49 ± 2.7%, *n* = 99) and Syt7 KO (20.7 ± 2.9%; mean ± SEM) cells, as was the mean number of fusion events per secreting cell (1.43 ± 0.26 in wild type vs. 1.63 ± 0.2, *n* = 75) in Syt7 KO). Further fusion occurred in wild type CTL after the change to 10 mM [Ca^2+^]. Remarkably, not a single fusion event was observed in Syt7 KO CTL upon change to 10 mM [Ca^2+^].

To test whether the absence of Syt7 caused the lack of fusion after the solution change in Syt7 KO cells, we re-introduced wild type Syt7 (via a Syt7-IRES-GFP construct) into Syt7 KO CTL by electroporation and quantified fusion in low [Ca^2+^] and then in 10 mM [Ca^2+^] solutions. Fusion in these CTL is shown in Figure 2A (Syt7 KO + Syt7). The number of fusion events per cell in low [Ca^2+^] was similar (1.22 ± 0.31, *N* = 3, *n* = 23) to wild type and Syt7 KO cells. However, expression of wild type Syt7 in Syt7 KO CTL restored fusion in the 10 mM [Ca^2+^] solution, with more fusion events per CTL (1.56 ± 0.39) than that observed in the wild type control, in high [Ca^2+^] solution (0.65 ± 0.18, *p* = 0.038), indicating that the lack of Syt7 prevents fusion events under these conditions. Cumulative plots of fusion events vs. time for wild type and Syt7 KO CTL and Syt7 expressing Syt7 KO CTL are shown in Figure 2B.

The fusion observed in the low [Ca^2+^] solution is likely supported by residual Ca^2+^ contained in the medium of harvested cells. We tested the extracellular free Ca^2+^ concentration of our low [Ca^2+^] solution using ratiometric recording of Fura-2ff fluorescence (see Materials and Methods). In nine solutions from three different preparations, the mean free [Ca^2+^] was 56 ± 5.8 μM.

The lack of fusion in Syt7 KO CTL in 10 mM [Ca^2+^] solutions could be due to differences in Ca^2+^ handling of wild type and Syt7 KO cells so we tested the cytosolic [Ca^2+^] of CTL under our recording conditions. The estimated mean cytosolic Ca^2+^ levels and change associated the switch to the 10 mM Ca^2+^ solution were similar in both groups (Figure 2C). The transient increases in intracellular [Ca^2+^] due to store release also had similar amplitudes and durations, as seen in representative traces from single cells (Figure 2D).

### Ca^2+^ Binding to Synaptotagmin7 Is Required for CG Replenishment at the IS

**Figure 3:**
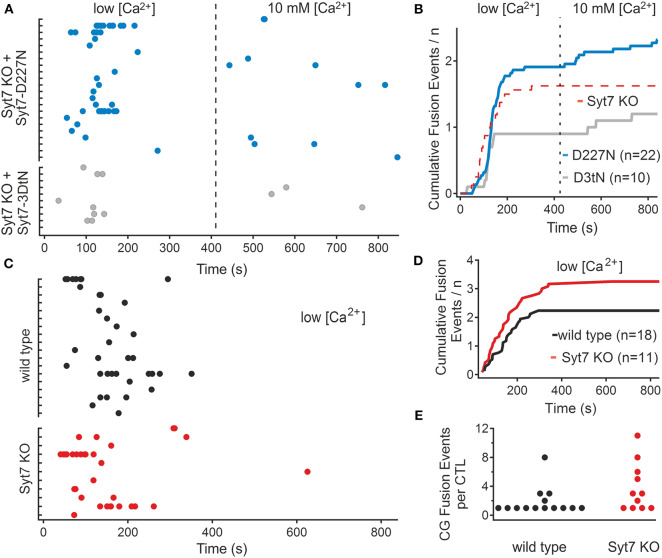
Mutation of Ca^2+^ binding aspartate residues to asparagine reduces the efficacy of rescue of fusion in 10 mM [Ca^2+^] in the Syt7 KO. **(A)** Scatter plots of fusion events for each CTL in Syt7 KO CTL rescued with either D227N Syt7 (*n* = 22) or the triple aspartate to asparagine mutant of the C2A domain (3DtN Syt7, *n* = 10). Expression of the D227N mutant (blue) produces similar fusion in low [Ca^2+^] to wild type CTL and supports fusion in 10 mM [Ca^2+^]. Expression of the 3DtN-Syt7 mutant in Syt7 KO CTL resulted in reduced overall fusion but still supported a late component of fusion. **(B)** Cumulative plots of all fusion events show that rescue with Syt7 D227N (blue) or Syt7 3DtN (gray) mutated C2A domains support fusion to a lesser degree than the wild type Syt7. The broken red line is the fusion of Syt7 KO cells shown as a reference. **(C)** Fusion of wild type (*n* = 18) and Syt7 KO (*n* = 11) CTL were observed in low calcium for 14 min. In the absence of a change to 10 mM calcium there was (with one exception) no additional fusion. **(D)** The cumulative fusion in wild type and Syt7 KO CTL was similar, with almost no fusion occurring after the first 7 min. **(E)** The fusion events per CTL were not significantly different in this experiment (Wilcoxon Rank test).

Ca^2+^ binding in synaptotagmins depends on the presence of aspartate residues in the C2 domains (5, 27). Ca^2+^ binding is augmented by additional charged amino acids and by negatively charged phospholipids in the associated membranes (28, 29), and there appears to be an interaction between the two C2 domains, referred to as C2A and C2B, to drive membrane fusion (30). In Syt7 the C2A is considered to play a greater role in Ca^2+^ sensing (31, 32). To test the role of Syt7 Ca^2+^ sensing in the restoration of secretion in 10 mM [Ca^2+^], we replaced an aspartate residue which contributes to Ca^2+^ binding in the C2A domain (33) of Syt7 with an asparagine (D227N). In a second set of experiments we replaced all three aspartates involved in Ca^2+^ binding of the C2A domain loop 3 of Syt7 with asparagine residues (3DtN: D225N, D227N, and D233N). We then recorded fusion in Syt7 KO cells expressing these two constructs. Figure 3A shows scatter plots of the fusion latencies of the CGs in Syt7 KO cells expressing either the single mutation (D227N, *N* = 3, *n* = 22) or Syt7 with all three aspartates of the C2A domain loop 3 (3DtN, *N* = 3, *n* = 10) mutated to asparagine. D227N expression supported fusion in the low [Ca^2+^] period and restored fusion events in 10 mM [Ca^2+^] in Syt7 KO CTL. The 3DtN mutants exhibited significantly fewer fusion events than the Syt7 expressing Syt7 KO CTL but still exhibited fusion in the 10 mM [Ca^2+^] solution. Figure 3B shows cumulative plots of fusion-events vs. time for each experimental group. In both groups fusion occurred with a short delay in low [Ca^2+^] and then ceased prior to the change to 10 mM [Ca^2+^]. The fusion in Syt7 KO CTL expressing either the D227N or 3DtN mutant was similar to that in wild type CTL or Syt7 KO CTL expressing Syt7 in low [Ca^2+^] but the D227N and 3DtN mutants had fewer fusion events/CTL than Syt7 expressing KO CTL (*p* = 0.023 and 0.038, respectively) in high [Ca^2+^].

To ensure that the fusion in high [Ca^2+^] was due to the increase in Ca^2+^ and not a delayed phase of fusion, the fusion of wild type and Syt7 KO cells was compared when maintained for 14 min in the low [Ca^2+^] solution. Fusion in WT and Syt7 KO cells was similar under these conditions (Figure 3C), with both groups exhibiting fusion preferentially in the first 3–4 min of the recording. Neither the fusion latency (Figure 3D) nor the numbers of CG fusion events per CTL (Figure 3E), were different (Wilcoxson's Rank test, *p* = 0.5) and with one exception, fusion did not occur in the second half of the recording consistent with a requirement for high [Ca^2+^] for the second phase of fusion.

**Figure 4:**
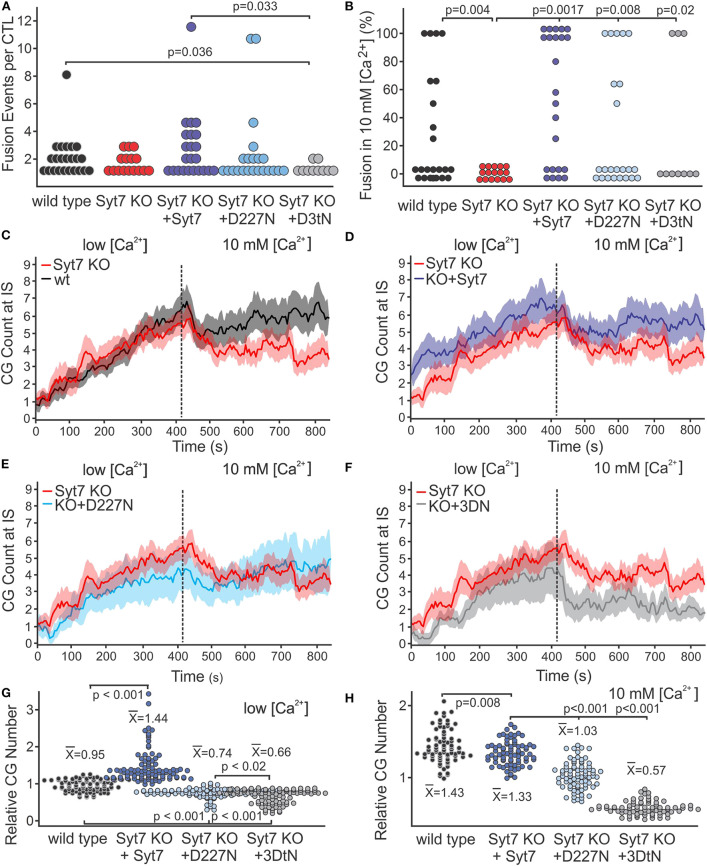
Syt7 affects the availability of CG at the IS. **(A)** Scatter plot of the number of fusion events per CTL is shown for all experimental groups. The 3DtN expressing KO cells exhibited significantly fewer fusion events per cell than wild type (*p* = 0.036) or Syt7 expressing Syt7 KO CTL (*p* = 0.033). **(B)** Syt7 is required for the restoration of fusion following addition of 10 mM [Ca^2+^]. Scatter plots of the percent of fusion events which occurred after addition of 10 mM [Ca^2+^] for each CTL in wild type, Syt7 KO and Syt7 KO expressing wild type Syt7 are shown. Both wild type and Syt7 KO CTL expressing wild type Syt7 exhibited secretion in 10 mM [Ca^2+^] (*p* = 0.004 and *p* = 0.0017, respectively). The CTL expressing D227N and 3DtN mutants also exhibited fusion events in 10 mM [Ca^2^] (*p* = 0.008 and *p* = 0.02, respectively, when compared to Syt7 KO CTL). **(C)** Lack of fusion events in 10 mM [Ca^2+^] is not due to a lack of CG at the IS. Traces show the CG visible at the IS during the recording periods (mean ± SEM) for wild type vs. Syt7 KO. **(D)** Syt7 KO CTL expressing wild type Syt7, **(E)** Syt7 KO CTL expressing the D227N mutant or **(F)** Syt7 KO CTL expressing the 3DtN mutant (all vs. Syt7 KO). **(G)** Scatter plot showing the CG number for wild type and rescue/CG number of Syt7 KO cells at the IS during the low [Ca^2+^] period. **(H)** Scatter plot showing the CG number for wild type and rescue/CG number of Syt7 KO cells at the IS during the high [Ca^2+^] period. The means of CG relative to Syt7 KO CG are shown (Wilcoxson Rank test).

Figure 4A shows the total fusion events per cell (combined low and high [Ca^2+^]) as scatter plots (wild type, black; Syt7 KO, red; Syt7 KO expressing Syt7, blue; Syt7 KO expressing D227N-Syt7, light blue; Syt7 KO expressing Syt7 3DtN gray, (mean ± SEM, black w/error bars). There were fewer fusion events per CTL in the 3DtN expressing KO CTL than in the wild type (*p* = 0.036) or Syt7 expressing KO CTL (*p* = 0.033), the only statistically significant differences among the groups). The fraction of Syt7 KO CTL secreting when expressing the D227N mutants was higher than that of wild type or Syt7 KO CTL, but the difference was not significant. The fraction of Syt7 KO cells expressing the 3DtN mutant which exhibited fusion events was similar to that of the wild type and Syt7 KO cells.

We compared the distribution of fusion events in low and high [Ca^2+^] among CTL by calculating the percent of total fusion events occurring in 10 mM [Ca^2+^] for each CTL. Scatter plots of the scores are shown in Figure 4B. The Syt7 KO cells did not show fusion in 10 mM [Ca^2+^]. The distributions of wild type (*p* = 0.004), Syt7 expressing Syt7 KO (*p* = 0.0017), D227N mutant expressing KO (*p* = 0.008) and 3DtN mutant (*p* = 0.02) CTL were significantly different compared to the Syt7KO CTL (Wilcoxson's Rank Test).

To determine if differences in numbers of fusion events could be explained by differences in granule availability, we counted the granules at the IS of the CTL used for the fusion experiments. In all conditions, CG number increased over time in the low [Ca^2+^] recording period (Figures 4C–F; mean ± SEM, vs. time, with the Syt7 KO (red) as reference). CG number in Syt7 KO cells was similar to that of the wild type cells, during the low [Ca^2+^] period. In Syt7 KO cells expressing wild type Syt7 the CG number was higher than in WT (*p* = 0.008) or Syt7 KO (*p* < 0.001) cells. The D227N and 3DtN mutants had similar granule numbers but had fewer granules at the IS than the wild type, Syt7 KO or the Syt7 KO expressing (*p* < 0.001 in each case). Thus, the expression of mutant Syt7 (especially the 3DtN mutant) in Syt7 KO CTL results in fewer CG at the IS than are observed in wild type or in Syt7 KO cells, possibly indicating a dominant negative affect.

After the change to 10 mM [Ca^2+^], all groups exhibited a rapid drop in granules at the IS followed by a plateau or recovery. This drop was not due to fusion, as it occurred before appreciable fusion and was present in the Syt7 KO cells and in the 3DtN expressing KO cells which showed no and little fusion, respectively, in 10 mM [Ca^2+^]. We have displayed the relative numbers of CG at the IS in low [Ca^2+^] (Figure 4G) and in 10 mM [Ca^2+^] (Figure 4H) as scatter plots. The points (granule counts/Syt7 KO granule counts) and the means ± SEM are shown for wild type and the three rescue groups. Scatter plots for the values for all traces and conditions are shown in Figure S3. Comparison of the granules at the IS among treatment groups indicates a lack of granules due to compromised trafficking does not fully explain the lack of fusion observed in 10 mM [Ca^2+^] by Syt7 KO cells. The availability of granules is lower in D227N (*p* < 0.001) and in the 3DtN (*p* < 0.001) compared to the rescue with wild type Syt7. Thus, granule availability is influenced by Syt7 and this affect appears to depend on Ca^2+^ binding.

The difference in availability of granules accounts for much of the deficit in 10 mM [Ca^2+^] fusion in the aspartate mutants. When the fusion events are corrected for differences in granule number, fusion in wild type, Syt7 KO and 3DtN rescued CTL was similar and much lower than fusion in Syt7 KO CTL expressing wild type Syt7. Fusion in 10 mM [Ca^2+^] appears Syt7-dependent, since Syt7 is required for the phase of fusion in 10 mM [Ca^2+^] and its deletion did not alter fusion in low [Ca^2+^]. The Syt7 KO expressing wild type Syt7 was the only group which exhibited more secretion in 10 mM [Ca^2+^] than in low [Ca^2+^].

### Synaptotagmin2 Might Be Involved in Ca^2+^-Dependent CG Fusion

**Figure 5:**
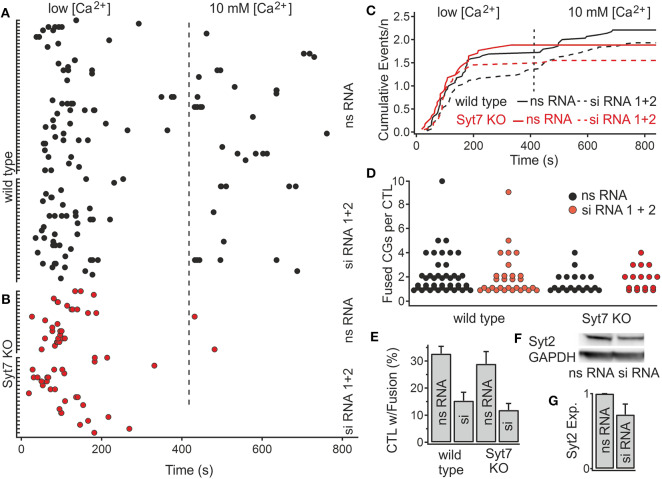
Reduction of Syt2 expression by siRNA application reduces the fraction of CTL exhibiting CG fusion events. **(A,B)** Scatter plot of the number of fusion events in wild type and Syt7 KO cells during low and high [Ca^2+^] conditions. The latency distributions of fusion events are unchanged following treatment with nonsense (ns RNA) or silencing (siRNA) treatment in **(A)** wild type or **(B)** Syt7 KO CTL. **(C)** Traces of cumulative secretion/n for the wild type and Syt7 KO CTL after treatment with nsRNA or siRNA show no significant differences. **(D)** The number of fusion events per CTL (exhibiting fusion) is not significantly altered following siRNA treatment. **(E)** The fraction of CTL exhibiting fusion events following siRNA treatment was reduced in wild type to 54% and Syt7 KO CTL to 61% of control (32.68–15.34%, *p* = 0.008, *n* = 4) in wild type cells and (28.95–11.9%, *p* = 0.005, *n* = 4) in Syt7 KO cells, Wilcoxson Rank test). **(F)** A representative Western blot showing Syt2 expression relative to GAPDH expression in wild type CTL following application of ns RNA or siRNA. **(G)** The average reduction in the expression of Syt2 by siRNA treatment was 29.8% (*n* = 3, *T*-test) when compared to ns RNA.

The above results indicate that Syt7 is not the Ca^2+^ sensor for CG fusion *per se*, as indicated by the lack of an effect in low [Ca^2+^]. As shown in Figure 1, Syt2 was detected in CTL and it is possible that Syt2, in spite of its low Ca^2+^ affinity, plays a role in CG fusion. We tested this possibility using a siRNA-mediated knock-down approach, since the Syt2 knock-out is prenatally lethal. We combined two different siRNAs which each led to a modest reduction in Syt2 signal in the time frame in which the experiments could be performed, and recorded fusion after electroporation of both siRNAs. Experiments in this time frame indicated that there was no change in the latency of fusion events in wild type (Figure 5A) or in Syt7 KO CTL (Figure 5B) that were treated with either non-silencing RNA or the silencing RNA. Cumulative plots of wild type CTL fusion and Syt7 KO CTL fusion following treatment with non-silencing (ns) RNA and silencing (si) RNA show that secretion is similar in all groups (Figure 5C). Though there was no significant difference in the fusion events per cell (Figure 5D), we observed a reduction in the fraction of cells in which fusion events occurred in wild type cells (Figure 5E) from 32.68 to 15.34 %, *p* = 0.008, *n* = 4) and in Syt7 KO cells (28.95 to 11.9% *p* = 0.005, *n* = 4, Students *T-*test). Western blots (Figure 5F) indicated that 24–30 h post-transfection there was a ~30% reduction in Syt2 protein levels in the CTL examined (Figure 5G). This result is modest but is consistent with a role for Syt2 in CG fusion.

## Discussion

We have examined the role of Syt7 in CG release in CD8^+^ CTL, based on its reported role in lysosomal fusion (13, 17), in CTL cytotoxicity and in clearance of infections (14). We show that both Syt7 and Syt2 RNA are present in activated CD8^+^ CTL. We were unable to demonstrate Syt7 in our activated CTL, but this may be due to low abundance, since a low number of granules are present in CTL, even when activated (34). We have observed a clear loss of fusion in the high calcium condition in the absence of Syt7. Syt7 KO CTL exhibited CG fusion similar to that of wild type CTL in low [Ca^2+^], indicating Syt7 is not required for CG fusion and thus may not cause primary immunodeficiencies. This is a novel result though it is not completely unexpected considering that Syt7 KO mice, although the immune response was compromised, could clear infections and that CD8^+^ CTL from Syt7 KO mice released granzyme A normally, in response to exposure to anti-CD3 antibodies (14). Though it was argued that activation using anti-CD3 antibodies may produce a stronger calcium signal and thus mask the role of Syt7. CTL killing is well-suited to function at low intra- and extracellular calcium levels. Our low [Ca^2+^] condition is within the range for efficient antigen-mediated cytotoxicity (35) and target cell killing has been demonstrated at extracellular calcium concentration as low as 100 μM (18). This result indicates that there is either a redundancy in the Ca^2+^ sensor for CG fusion or that a calcium sensor is not required at all.

Syt7 KO CTL exhibit normal numbers of CG and can deliver these granules to the IS after activation (14). Our results in low calcium are consistent with this finding. There were no differences in CG counts between wild type and Syt7 KO CTL in the low calcium condition. Syt7 KO CTL expressing wild type Syt7 had modest increases in granule number. Only when challenged with the high calcium condition, which produces a second phase of CG fusion, did the lack of Syt7 produce a phenotype which includes lower granule counts and a lack of fusion.

Expression of wild type Syt7 in the Syt7 KO CTL rescued granule counts and fusion in high calcium. Though C2A domain aspartate mutants reduced granule numbers they did produce fusion events in 10 mM [Ca^2+^]. Replacement of a single aspartate with asparagine in the C2B domain of Syt can lead to an apparent increase in Ca^2+^ sensitivity (29, 36, 37) while replacement of all three aspartates of loop 3 causes a strong reduction in evoked/and or spontaneous release (29, 32, 36, 38, 39). Though there is a preference for either the C2A or C2B domain, depending on which Syt is tested, C2A and C2B domains act in tandem (30, 40, 41). In our hands the single aspartate mutation in the C2A domain produced a reduction in CG number in the TIRF field without reducing fusion events per CTL, in comparison to wild type and Syt7 KO cells expressing wild type Syt7. The triple mutation led to a reduction in CGs in the TIRF field and strongly reduced fusion in 10 mM [Ca^2+^], when compared to the rescue with wild type Syt7.

CTL typically only release a small fraction of the available granules and this appears to be the case under our conditions. Both phases of fusion lasted only a few minutes and fusion ended in spite of the continued presence of CGs. The lack of fusion in 10 mM [Ca^2+^] in the Syt7 KO and its rescue following expression of wild type Syt7 in the Syt7 KO CTL indicate that Syt7 is required for the second phase of fusion. The low granule counts, in particular in the high calcium condition, in the Syt7 KO indicate that Syt7 is involved in the replenishment or maintenance of CG at the IS.

Fusion after the change to high calcium medium required the high calcium condition, based on the control experiments done in continuous low calcium. The poor performance of the triple mutant in 10 mM [Ca^2+^] can only be attributed in part to the lower granule counts. The very low fusion of the aspartate mutants may indicate that under these conditions Syt7 is involved in priming or fusion of CGs, and Syt7 clearly plays a permissive role in fusion in high calcium. Synaptotagmins are thought to be a part of a docking complex which promotes generation of SNARE complexes (4) required for priming. Syt7 has been reported to play a number of other roles in a variety of cell types, including a role in vesicle trafficking (26).

The knock-down of Syt2 in wild type and Syt7 KO cells produced equivocal results. There was no change in the latency of fusion. The number of fusion events per cell was slightly increased but this change was not significant, which might be interpreted as evidence for no role of Syt2 in fusion. The reduction in the cells exhibiting fusion events was highly significant, however, and may be a hint the Syt2 is involved. Though its Ca^2+^ affinity is low, Ca^2+^ sensors with low affinity have been reported to support spontaneous and/or asynchronous release of neurotransmitter under low [Ca^2+^]_i_ conditions in neurons (38, 42, 43). Syt2 has been reported to be involved in regulated exocytosis from mast cells (44) and in phagocytosis in neutrophils (45). In addition, there are other potential Ca^2+^ sensors such as the DOC2 family (46) which have also been suggested to support spontaneous or asynchronous release under low [Ca^2+^]_i_ conditions. Our results neither confirm nor rule out a role for Syt2. Additional Syt2 knock-down or Syt2 knock-out experiments will be required to address this question.

Taken together, our results indicate that Syt7 is not required for CG fusion *per se*. The change in granule numbers following expression of Syt7 in the Syt7 knock-out indicates that Syt7 is involved in trafficking of CGs. The loss of fusion events in the high calcium condition in Syt7 KO CTL and their rescue with Syt7 cannot be completely explained by reductions in CG availability and indicate an additional role for Syt7 in fusion which requires more study.

## Data Availability Statement

All datasets generated for this study are included in the article/Supplementary Material.

## Ethics Statement

The animal study was reviewed and approved by Saarland University.

## Author Contributions

MS, PC, and DS performed experiments, analyzed results, and made the figures. DS and JR designed the research and wrote the paper.

## Conflict of Interest

The authors declare that the research was conducted in the absence of any commercial or financial relationships that could be construed as a potential conflict of interest.

## Supplementary Material

Figure S1 **(A)** Western blot showing the expression of Syt2 in wild type and Syt7 KO CTL on the third day of activation. GADPH bands are shown as a loading control. **(B)** The relative density of the Syt2 expression is shown based on the density of the GAPDH loading controls bands (mean ± SEM, *n* = 3).

Figure S2 Identification of fusion events using TIRF imaging. **(A)** A sequence of video frames showing granzyme B-mCherry fluorescence of a single granule over time. Fluorescence is measured at three regions of interest. The blue circle contains the granule. A second ROI (red) is placed adjacent to the granule and a third (purple) is further away. The frame rate is 10 Hz. The granule loses brightness upon fusion. As the fluorophore diffuses the fluorescence increases at the adjacent ROI and later at the distant ROI. (depicted in the 2 s frame). **(B)** The fluorescence at the three ROIs depicted in **(A)** charted (color coded) vs. time, showing the increase in fluorescence due to diffusion of the fluorescent cloud which is delayed, in particular at the more remote ROI. **(C)** Two sets of representative images depicting CG in the TIRF field. In both cells several granules are present and in each cell one granule (yellow arrow) undergoes fusion at 0.4 s.

Figure S3 Scatter plots of data for mean granule counts. **(A)** Data points for all five treatment groups for the entire experiment are shown. **(B)** Data points for the low Ca^2+^ period for all five treatment groups are shown. **(C)** Data points for all treatment groups during the 10 mM Ca^2+^ treatment period are shown. The mean for each group is shown as a gray bar. The statistical significance of differences between treatment groups were established using the Wilcoxon Rank test following a one-way ANOVA test. *P*-values lower than 0.001 are shown as ^***^. Non significance is indicated as ns.

Table S1 List of primers, amplified exons, annealing temperatures, and expected PCR product sizes.
