# Supplementary figures and images for: Cytotoxic Granule Trafficking and Fusion in Synaptotagmin7-Deficient Cytotoxic T Lymphocytes
Source: Front Immunol. 2020 May 29;11:1080. doi: 10.3389/fimmu.2020.01080 (PMC7273742; doi:10.3389/fimmu.2020.01080)

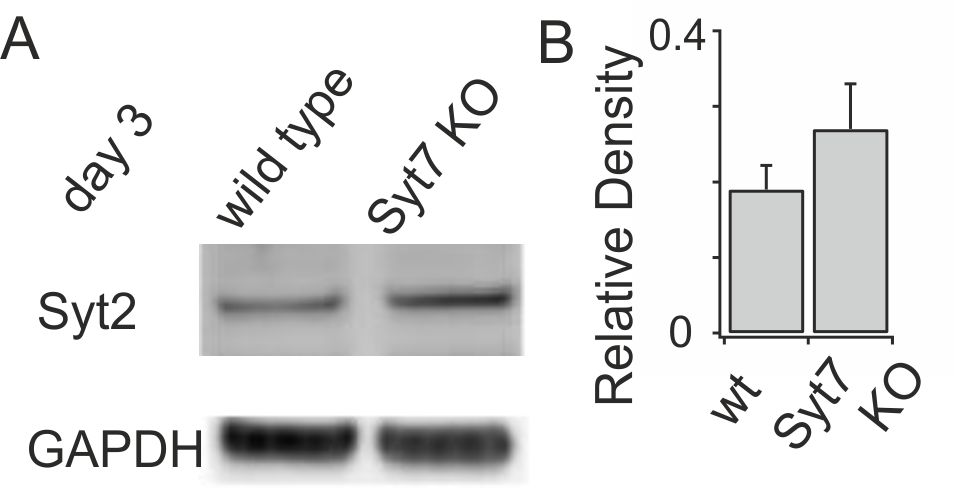

Supplement: Figure S1 — (A) Western blot showing the expression of Syt2 in wild type and Syt7 KO CTL on the third day of activation. GADPH bands are shown as a loading control. (B) The relative density of the Syt2 expression is shown based on the density of the GAPDH loading controls bands (mean ± SEM, n = 3). [file Image_1.JPEG]

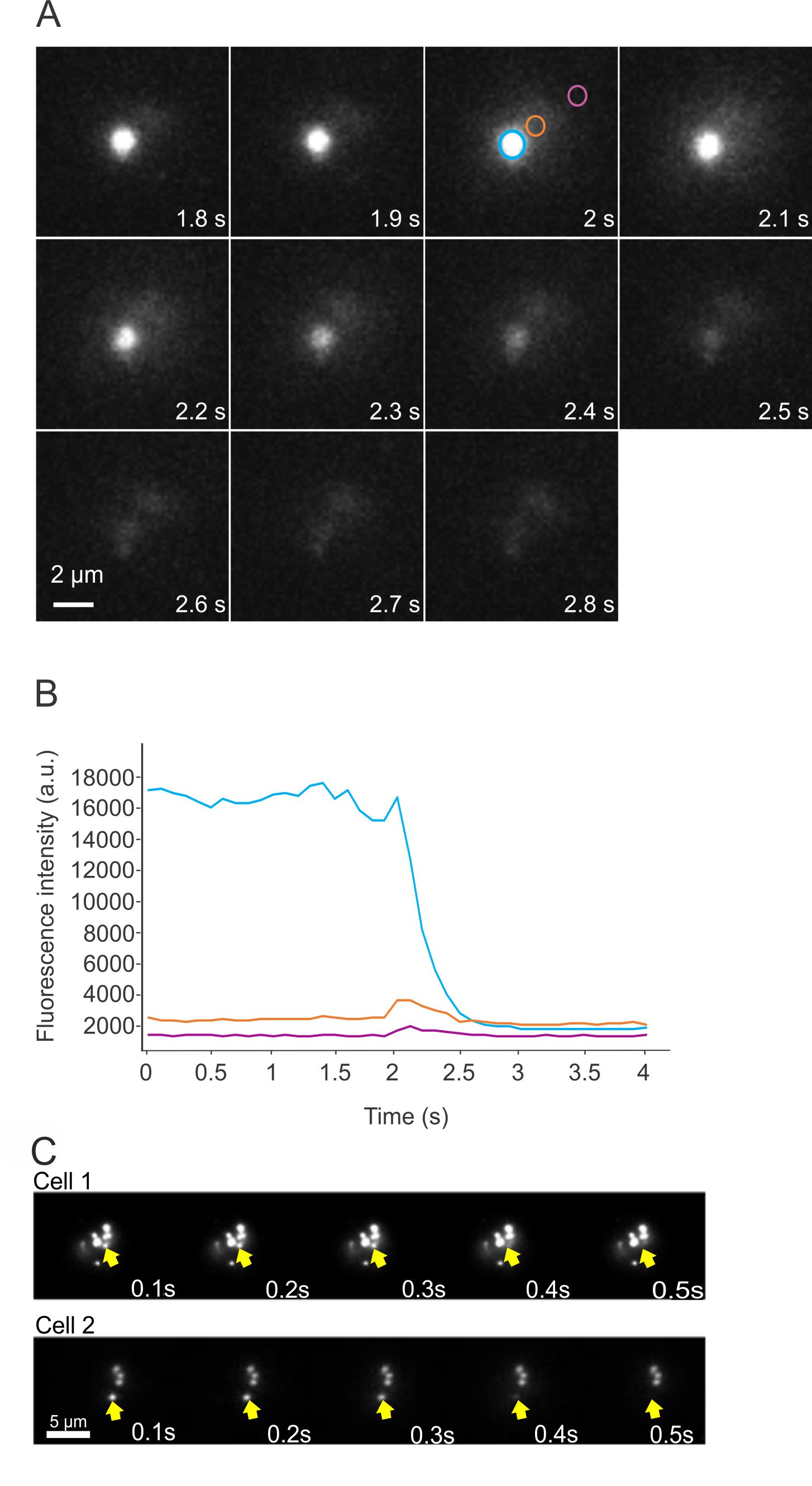

Supplement: Figure S2 — Identification of fusion events using TIRF imaging. (A) A sequence of video frames showing granzyme B-mCherry fluorescence of a single granule over time. Fluorescence is measured at three regions of interest. The blue circle contains the granule. A second ROI (red) is placed adjacent to the granule and a third (purple) is further away. The frame rate is 10 Hz. The granule loses brightness upon fusion. As the fluorophore diffuses the fluorescence increases at the adjacent ROI and later at the distant ROI. (depicted in the 2 s frame). (B) The fluorescence at the three ROIs depicted in (A) charted (color coded) vs. time, showing the increase in fluorescence due to diffusion of the fluorescent cloud which is delayed, in particular at the more remote ROI. (C) Two sets of representative images depicting CG in the TIRF field. In both cells several granules are present and in each cell one granule (yellow arrow) undergoes fusion at 0.4 s. [file Image_2.JPEG]

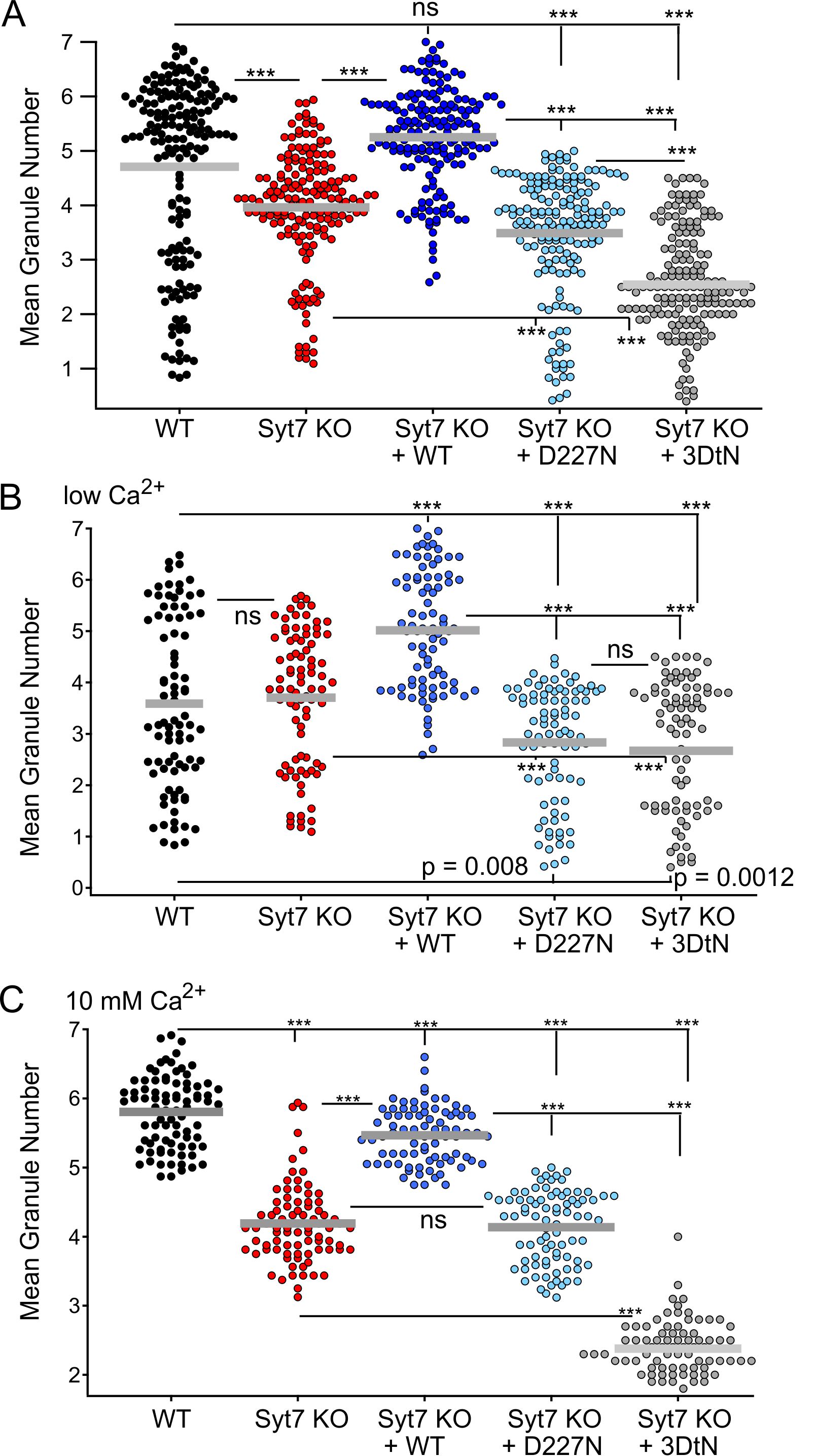

Supplement: Figure S3 — Scatter plots of data for mean granule counts. (A) Data points for all five treatment groups for the entire experiment are shown. (B) Data points for the low Ca2+ period for all five treatment groups are shown. (C) Data points for all treatment groups during the 10 mM Ca2+ treatment period are shown. The mean for each group is shown as a gray bar. The statistical significance of differences between treatment groups were established using the Wilcoxon Rank test following a one-way ANOVA test. P-values lower than 0.001 are shown as ***. Non significance is indicated as ns. [file Image_3.JPEG]
