# Supplementary material for: Cytotoxic Granule Trafficking and Fusion in Synaptotagmin7-Deficient Cytotoxic T Lymphocytes
Source: Front Immunol. 2020 May 29;11:1080. doi: 10.3389/fimmu.2020.01080 (PMC7273742; doi:10.3389/fimmu.2020.01080)
Supplement: Table S1 — List of primers, amplified exons, annealing temperatures, and expected PCR product sizes. [file Data_Sheet_1.docx]

**Table S1: List of primers, amplified exons, annealing temperatures, and expected PCR product sizes.**
